# Presidential Symposium: The Future of Aging Equity: Sustaining Progress and Innovating Inclusion

**DOI:** 10.1093/geroni/igaf122.1092

**Published:** 2025-12-31

**Authors:** Chivon Mingo

## Abstract

Aging is a fundamental aspect of identity and lived experience—yet it is often overlooked when considering equity across society. How age intersects with race, socioeconomic status, disability, LGBTQ+ identity, and geography shapes opportunities, access to care, and overall well-being throughout the lifespan. Ensuring that all individuals can age with dignity, health, and security requires a commitment to aging equity (i.e., the fair and just distribution of opportunities, resources, and support for older adults). As research, policy, advocacy, and practice efforts continue to push for inclusion, shifting funding priorities and institutional frameworks introduce new complexities that challenge the sustainability of progress. While new barriers continue to emerge, the need to sustain and advance aging equity has never been more urgent. This symposium brings together experts in policy, research, and applied practice to explore key strategies for securing the future of aging equity. Speakers will highlight how we can sustain hard-fought gains while forging new paths—leveraging innovation, collaboration, and research to drive inclusion, even in uncertain times. This session takes a firm stance on sustaining and expanding equity efforts across these intersecting dimensions of identity while developing forward-thinking strategies to ensure continued progress despite evolving challenges.

